# Prognostic significance of STAT3 and phosphorylated STAT3 in human soft tissue tumors - a clinicopathological analysis

**DOI:** 10.1186/1756-9966-30-56

**Published:** 2011-05-16

**Authors:** Diana David, Lakshmy M Rajappan, Krishna Balachandran, Jissa V Thulaseedharan, Asha S Nair, Radhakrishna M Pillai

**Affiliations:** 1 Integrated Cancer Research, Rajiv Gandhi Centre for Biotechnology, Kerala, India; 2 District Public Health Laboratory, Alappuzha, Kerala, India; 3 Department of Pathology, Kottayam Medical College, Kottayam, Kerala, India

**Keywords:** STAT3, pSTAT3, Soft tissue tumors

## Abstract

**Background:**

Signal transducer and activator of transcription 3 (STAT3) is a key signaling molecule and a central cytoplasmic transcription factor, implicated in the regulation of growth. Its aberrant activation has been demonstrated to correlate with many types of human malignancy. However, whether constitutive STAT3 signaling plays a key role in the survival and growth of soft-tissue tumors is still unclear and hence needs to be elucidated further. In our study we examined the expression levels of STAT3 and pSTAT3 in different grades of soft tissue tumors and correlated with its clinicopathological characteristics.

**Methods:**

Expression levels of STAT3 and pSTAT3 in soft tissue tumors were studied using Immunohistochemistry, Western blotting and Reverse transcriptase- PCR and correlated with its clinicopathological characteristics using Chi squared or Fisher's exact test and by logistic regression analysis. Statistical analysis was done using Intercooled Stata software (Intercooled Stata 8.2 version).

**Results:**

Of the 82 soft tissue tumor samples, fifty four (65.8%) showed immunoreactivity for STAT3 and twenty eight (34.1%) for pSTAT3. Expression of STAT3 and pSTAT3 was significantly associated with tumor grade (P < 0.001; P < 0.001), tumor location (P = 0.025; P = 0.027), plane of tumor (P = 0.011; P = 0.006), and tumor necrosis (P = 0.001; P = 0.002). Western blotting and RT-PCR analysis showed increased expression of STAT3 and p-STAT3 as grade of malignancy increased.

**Conclusion:**

These findings suggest that constitutive activation of STAT3 is an important factor related to carcinogenesis of human soft tissue tumors and is significantly associated with its clinicopathological parameters which may possibly have potential diagnostic implications.

## Background

STATs comprise a family of seven proteins (STAT 1, 2, 3, 4, 5a, 5b, and 6) unique in their ability both to transduce extracellular signals and regulate transcription directly [1]. STAT3 normally resides in the cytoplasm and is often constitutively activated in many human cancer cells and tumor tissues and has been shown to induce expression of genes involved in cell proliferation and survival [2,3]. Constitutively activated STAT3 correlates with a more malignant tumor phenotype, resistance to chemotherapy and is also associated with decreased survival in some cancers [4,5]. Recently, STAT3 has been implicated as a promising target for therapeutic intervention in cancer [6].

Soft tissue tumors comprise of a group of relatively rare, anatomically and histologically diverse neoplasms derived from tissues of mesodermal and ectodermal layer. Clinically, soft tissue tumors range from totally benign to highly malignant neoplasms. Many are of an intermediate nature, which typically implies aggressive local behavior with a low to moderate propensity to metastasize. The incidence of soft tissue tumors is low accounting for 1% of adult malignancies and 15% of pediatric malignancies [7]. Mortality, on the other hand, is high; the average five-year survival rate is only 60%. Most soft tissue tumors arises de novo, but a small number originates in injured tissue such as scars or radiation-exposed areas [8]. Sarcomas possess specific molecular characteristics and frequently present distinct diagnostic problems, and even many of the better-characterized tumors still lack reliable prognostic markers. New specific molecular genetic markers are expected to become increasingly useful in the clinical evaluation of such tumors [9].

Considering the important role of STAT3 and pSTAT3 in various cancers, our study aimed to analyze the expression levels of STAT3 and pSTAT3 in soft tissue tumors by Immunohistochemistry, Western blotting and RT-PCR. In addition we compared STAT3 and pSTAT3 expression with clinicopathologic parameters of soft tissue tumors.

## Methods

### Patients and specimens

Primary surgical specimens were obtained from 82 patients (51 males and 31 females) who were clinically diagnosed for soft tissue tumors, from Department of General Surgery, Govt. Medical College Hospital, Thiruvananthapuram, India between 2007 and 2008 following approval from the Human Ethics Committee. Of the 82 cases, 48 were malignant, 25 benign, and 9 were of intermediate grade. Tumor stages were classified according to the revised GTNM (grade-tumor-node-metastasis) classification of WHO (2002).

### Histopathologic examination of soft tissue tumors

The present study correlated the gross pathological features of soft tissue tumors like tumor size, location, depth, circumscription, encapsulation and presence of necrosis with clinical parameters. Histopathological parameters were studied using 5 μm thick paraffin sections stained with Hematoxylin and Eosin and the tumors were broadly classified into benign, intermediate and malignant.

### Immunohistochemistry and evaluation

Resected specimens were fixed with 10% paraformaldehyde and embedded in paraffin blocks. Five-micrometer sections of 82 representative soft tissue tumor blocks were used for immunohistochemical analysis. Sections were deparaffinized in xylene and rehydrated in graded alcohols and water. Endogenous peroxidase activity was blocked via treatment with 2.5% hydrogen peroxide for 20 minutes. Antigen retrieval was performed by placing the slides in boiling citric acid buffer (10 mM sodium citrate and 10 mM citric acid) for 15 minutes. Sections were treated with protein-blocking solution for 30 minutes and primary antibodies such as STAT3 and pSTAT3 (Santa Cruz Biotechnology, Inc, CA) were applied at a 1:100 and 1:50 dilution and incubated overnight at 4°C. After several rinses in phosphate-buffered saline, the sections were incubated in biotinylated secondary antibody for 30 minutes. The bound antibodies were detected by a streptavidin-biotin method, with a Vecta Elite ABC staining kit (Vector Laboratories). The slides were rinsed in phosphate-buffered saline, exposed to diaminobenzidine, and counterstained with Mayer's hematoxylin. For the tumor tissues, nuclear STAT3 and pSTAT3 (Tyr 705) staining were recorded as the numbers of STAT3 and pSTAT3-positive nuclei, divided by the total number of nuclei of at least 10 fields, and then expressed as a percentage. Cytoplasmic positivity of STAT3 and pSTAT3 were measured depending on the intensity of immunoreactivity (independently scored by D.D, AN, and LMR) and scored as mild (+), moderate (++), and intense (+++).

### Immunoblot analysis

Protein extracts were prepared by homogenizing fresh tissue in lysis buffer comprising 10% NP40, 5 M NaCl, 1 M HEPES, 0.1 M DTT, 0.1 M EGTA, 0.1 M EDTA, protease inhibitors (Sigma) and differential centrifugation (14000 rpm for 10 minutes). The protein concentrations were determined using Bradford's assay and 60 μg of proteins were resolved by 10% SDS-PAGE, and the separated proteins were electrotransferred onto nitrocellulose membrane (Amersham Pharmacia Biotech). After preblocking these membranes with 5% skimmed milk, they were treated with antibodies against STAT3 (1:200, Santa Cruz Biotechnology), pSTAT3 (Tyr 705) (1:200, Santa Cruz Biotechnology), and β- actin (1:5000, Sigma) as primary antibodies and incubated overnight at 4ºC. Horseradish peroxidase-conjugated antirabbit (1:5000, Santa Cruz Biotechnology) and antimouse (1:5000, Santa Cruz Biotechnology) antibodies were used as secondary antibodies and incubated for 1 h at room temperature. Immunoreactive bands were developed with an ECL system (Amersham Pharmacia Biotech, Uppsala, Sweden).

### Reverse Transcription - PCR

Total RNA was isolated from fresh tissues using TRIzol (Invitrogen) reagent. 10μg of total RNA was converted to cDNA using M-MLV Reverse Transcriptase (Promega) in a 25μl reaction. The relative expression of STAT3 was analyzed using semi-quantitative reverse transcription- PCR with glyceraldehyde-3-phosphate dehydrogenase (GAPDH) as an internal control. The primers used were STAT3 (sense), 5'-GGAGGAGTTGCAGCAAAAAG-3'; STAT3 (antisense) 5'-TGTGTTTGTGCCCAGAATGT-3'; GAPDH (sense), 5'-TTGGTATCGTGGAAGGACTCA-3'; GAPDH (antisense), 5'-TGTCATCATATTTGGCAGGTT-3'.The RT-PCR reaction mixture contained 5μl of 10× reaction buffer, 5μl of cDNA template, 0.5 μL each of forward and reverse primers, and 0.5 μL of Dr Taq DNA polymerase (Biogene) in a final volume of 50 μL. The reaction was done at 94°C for 4 min (Initial denaturation), 94°C for 30 s (Denaturation), 60°C for 40 s (Annealing), 72°C for 1 min and 30 s (Extension), and 72°C for 7 min (Final extension) for 35 cycles. Analysis of amplified products was done on 2% agarose gel and visualized using Fluor-S™ MultiImager (Bio-Rad). The PCR products were quantified by densitometric analysis, using Bio-Rad Quantity One software. The mRNA levels of STAT3 were normalized to human GAPDH mRNA levels. A 100-bp ladder was used as a size standard.

### Statistical analysis

Statistical analysis was performed using Intercooled Stata software (Intercooled Stata 8.2 version). The clinicopathological characteristics of the patients were compared between tumor grade, and expression of STAT3 and pSTAT3, using Chi squared or Fisher's exact test. The limit of statistical significance was set at P < 0.05. The effect of clinicopathologic characteristics on STAT3 and pSTAT3 expression were estimated with Odds Ratio (OR) and their 95% Confidence Interval (CI) derived from logistic regression analysis. Sensitivity and specificity of STAT3 and pSTAT3 expression were determined by taking the histopathological grade of tumor as the Gold standard.

## Results

### Clinicopathological characteristics of soft tissue tumors

The patients included in this study were aged from 1 to 80 years (Mean 42, SD = 19.8). Both age and sex of the patients showed significant association with tumor grade (P = 0.012; P = 0.04). Tumor size and tumor location also showed significant association with grade of the tumor (P = 0.004; P = 0.009). While most of the benign tumors occurred in the extremities (68%), the lower extremities (45.8%) followed by the retroperitoneum (27.1%) were the favored sites for malignant tumors. Tumors of intermediate grade were more common in the trunk (55.6%). Most of the soft tissue tumors in the present study were located in the subcutaneous plane (52.4%) followed by the muscular plane (28%).

**Figure 1:**
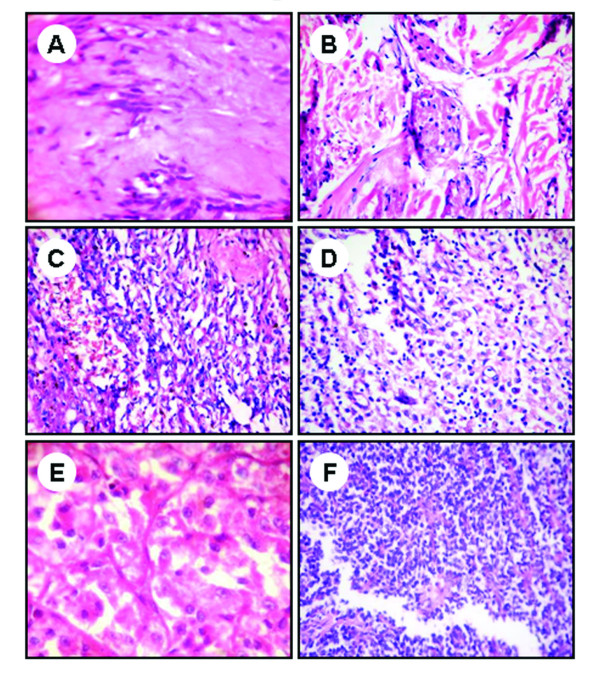
**Pathologic features of benign, intermediate, and malignant soft tissue tumors. Benign tumor (A) shows cystic degeneration and nuclear palisading and (B) shows nests of granular cells separated by fibrocollagenous tissue**. The intermediate grade tumors (C) shows solid, cellular lobules consisting of plump endothelial cells lining tiny rounded vascular spaces with inconspicuous and (D) shows proliferation of spindle cells in inflammatory background. The malignant soft tissue tumors (E) shows epithelioid cells arranged in nests, with a pseudoalveolar pattern and (F) shows lobulated vascular neoplasm composed of small blue round cells in sheets and rosettes. Image magnifications are 400×.

Among the 82 tumors studied, 38 were well-circumscribed and showed significant association with tumor grade (P < 0.001). Necrosis was studied in all the tumors and significant association was observed with the grade of the tumor (P < 0.001). Tables 1 list the clinicopathological characteristics of the soft tissue tumors selected for the study. Pathologic features of the representative benign, intermediate and malignant soft tissue tumors were given in Figure 1.

**Table 1:** Clinicopathologic characteristics of soft tissue tumors

Characteristics	Grade of tumor				
	
	Benign	Intermediate	Malignant	Total	P- value
**Number of patients**	25(100)	9(100)	48(100)	82(100)	
**Sex**					
Male	16(64)	2(22.2)	33(68.7)	51(62.2)	0.04
Female	9(36)	7(77.8)	15(31.3)	31(37.8)	
					
**Age**					
< 20	6(24)	0(0)	7(14.6)	13(15.8)	0.012
20-39	7(28)	6(66.7)	8(16.7)	21(25.6)	
40-59	9(36)	0(0)	21(43.7)	30(36.6)	
> = 60	3(12)	3(33.3)	12(25)	18(21.9)	
					
**Tumor size**					
< = 5 cm	16(64)	2(22.2)	13(27.1)	31(37.8)	0.004
>5 & < = 10 cm	7(28)	3(33.3)	12(25)	22(26.8)	
>10 & < = 15 cm	0(0)	4(44.4)	11(22.9)	15(18.3)	
>15 & < = 20 cm	2(8)	0(0)	7(14.6)	9(11)	
>20 cm	0(0)	0(0)	5(10.4)	5(6.1)	
					
**Tumor location**					
Upper limb	8(32)	0(0)	5(10.4)	13(15.8)	0.009
Lower limb	9(36)	4(44.4)	22(45.8)	35(42.7)	
Thorax	6(24)	5(55.6)	7(14.6)	18(21.9)	
Head & neck	1(4)	0(0)	1(2.1)	2(2.4)	
Retroperitoneum	1(4)	0(0)	13(27.1)	14(17.1)	
					
**Plane of tumor**					
Subcutis	21(84)	6(66.7)	16(33.3)	43(52.4)	< 0.001
Muscular plane	3(12)	3(33.3)	17(35.4)	23(28.0)	
Body cavity	1(4)	0(0)	15(31.2)	16(19.5)	
					
**Circumscription**					
No	5(20)	7(77.8)	32(66.7)	44(53.7)	< 0.001
Yes	20(80)	2(22.2)	16(33.3)	38(46.3)	
					
**Capsulation**					
No	20(80)	9(100)	44(91.7)	73(89.0)	0.232
Yes	5(20)	0(0)	4(8.3)	9(11)	
					
**Necrosis**					
No	25(100)	7(77.8)	29(60.4)	61(74.4)	< 0.001
Yes	0(0)	2(22.2)	19(39.6)	21(25.6)	

### Immunohistochemistry for STAT3 and pSTAT3

#### Overexpression of STAT3 and p-STAT3 correlates with tumor grade

**Figure 2:**
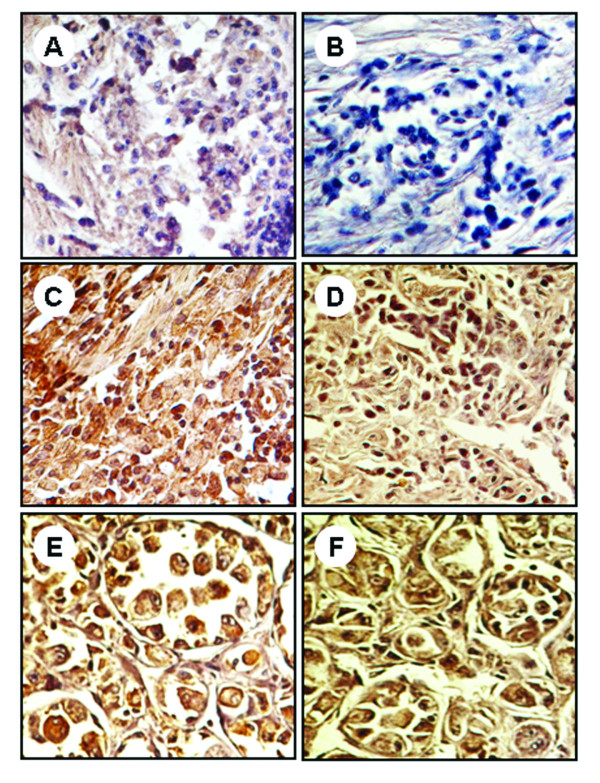
**Expression of immunohistochemical markers, STAT3 (A, C, E) and p-STAT3 (B, D, F), in benign (A and B); intermediate (C and D); malignant (E and F) soft tissue tumors**. The nuclei were counterstained with hematoxylin blue. Image magnifications are 400×.

Immunohistochemical staining revealed both cytoplasmic and nuclear localization of STAT3 and pSTAT3 in benign, intermediate, and malignant soft tissue tumors [Figure 2]. Two of 25 benign tumors expressed mild cytoplasmic positivity for STAT3 whereas 6 intermediate tumors exhibited both mild and moderate cytoplasmic positivity for STAT3. Thirty seven of the 46 malignant tumors showed intense STAT3 expression in the cytoplasm whereas the remaining 9 tissues showed moderate and mild cytoplasmic positivity. pSTAT3 expression was not observed in benign tumors. Both mild and moderate cytoplasmic expression of pSTAT3 was observed in intermediate tumors and only malignant tumors exhibited intense cytoplasmic expression for pSTAT3.

The percentages of positive nuclear expression of STAT3 and pSTAT3 in benign, intermediate, and malignant soft tissue tumors were also analyzed. The intermediate tumors expressed 52% nuclear expression for STAT3 while this was 85% in malignant tumors. Nuclear expression of pSTAT3 in intermediate and malignant tumors was 47% and 60% respectively. Nuclear expression of STAT3 and pSTAT3 were not observed in benign soft tissue tumors. Tables 2 lists and summarize the percentages of expressed STAT3 and pSTAT3 in all tumor groups.

**Table 2:** Expression levels of STAT3 and pSTAT3 in benign, intermediate and malignant human soft tissue tumors.

	STAT3				pSTAT3			
	**Cytoplasm n (%)**		**Nucleus n (%)**		**Cytoplasm n (%)**		**Nucleus n(%)**	
	
		**Mild (+)**	**Moderate (++)**	**Intense(+++)**		**Mild (+)**	**Moderate (++)**	**Intense(+++)**
				
**Benign**(n = 25)	2(8)	0(0)	0(0)	0(0)	0(0)	0(0)	0(0)	0(0)
**Intermediate**(n = 9)	2(8)	4(44.4)	0(0)	5(55)	3(33.3)	1(11.1)	0(0)	4(44)
**Malignant**(n = 48)	2(8)	7(14.6)	37(77.1)	42(87.5)	7(14.6)	12(25)	5(10.4)	24(50)

### Immunoblot analysis of STAT3 and pSTAT3 in soft tissue tumors

#### STAT3 and p-STAT3 are constitutively expressed in soft tissue tumors

**Figure 3:**
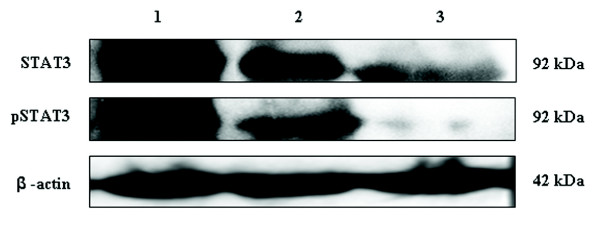
**Representative Western blotting analysis of STAT3 and pSTAT3 in soft tissue tumor extracts. Increased expression of STAT3 and pSTAT3 were observed in high and intermediate grade soft tissue tumors compared to benign tumors**. Lane 1: malignant soft tissue tumor; lane 2: intermediate soft tissue tumor; lane 3: benign soft tissue tumor. β-actin was used to verify equal gel loading.

The expression levels of STAT3 and pSTAT3 were analyzed by immunoblotting in representative soft tissue tumor samples [Figure 3]. STAT3 was found to be overexpressed in malignant tumors, when compared with intermediate and benign soft tissue tumors. The malignant tumor samples showed high level expression of pSTAT3 when compared with intermediate and benign soft tissue tumors. The data also revealed that STAT3 and pSTAT3 band intensities correlated to immunohistochemistry results.

### Expression of STAT3 at the mRNA level in soft tissue tumors

#### STAT3 gene expression correlates with tumor grade in soft tissue tumors

**Figure 4:**
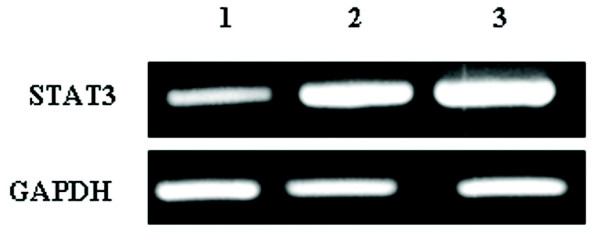
**Representative ethidium bromide stained 2% agarose gel showing semiquantitative Reverse Transcriptase polymerase chain reaction (RT-PCR) analysis and quantification of STAT3 (298 bp) mRNA expression at different stages of soft tissue tumors v/s GAPDH (269 bp) (A and B)**. Lane 1: benign soft tissue tumor; lane 2: intermediate soft tissue tumor; lane 3: malignant soft tissue tumor. A 100-bp ladder was used as a size standard.

**Figure 5:**
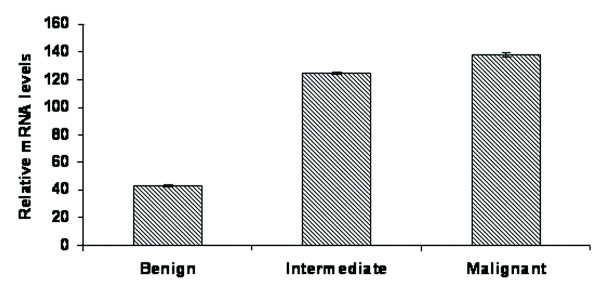
**The mRNA levels of STAT3 were normalized to human GAPDH mRNA levels and was analyzed by Spearman's rank correlation coefficient which gives a value of Spearman's rho (*ρ*) = 1, and p-value < 0.001, indicating a significant positive correlation**. Bar graph shows mean value ± S.E. from three independent experiments.

Reverse transcription -PCR was done to analyze the mRNA level expression of STAT3 in representative soft tissue tumor samples [Figure 4]. A high level expression of STAT3 mRNA was observed in tumor samples. Among the tumor samples, STAT3 mRNA was found to be overexpressed in malignant and intermediate tumors when compared with benign soft tissue tumors [Figure 5]. Together these results indicate that fluctuations observed in STAT3 mRNA expression correlated with its protein level expression.

### Statistical analysis

Expression of STAT3 and pSTAT3 showed statistically significant association with histopathological parameters as evidenced by Chi squared and Fisher's exact test [See Additional file 1 Table S1]. STAT3 and pSTAT3 expressions were significantly associated with grade of the tumor (P < 0.001). Malignant tumors were 107.3 times more likely to express STAT3 (OR = 107.3, 95% CI: 20.24-569), and 7.5 times more likely to express pSTAT3 (OR = 7.5, 95% CI: 2.28-24.5) when benign or intermediate tumor is the reference [Table 3]. The sensitivity and the specificity of STAT3 were 95.8% and 76.5% and pSTAT3 were 50% and 88.2%, respectively, with histopathological grade. In addition, Table 4 represents the association between clinicopathologic characteristics and expression of STAT3 in malignant soft tissue tumors.

**Table 3:** Univariate logistic regression analysis: Significant association between expression of STAT3 and pSTAT3 and clinicopathological characteristics of soft tissue tumors.

Clinicopathological characteristics	STAT3			pSTAT3		
	
	OR	95% CI	P-value	OR	95% CI	P-value
**Grade of tumor**						
Benign or intermediate	1			1		
Malignant	107.3	20.24-569	< 0.001	7.5	2.28-24.5	0.001
						
**Tumor Size**						
< = 5 cm	1			1		
>5 & < = 10 cm	2.42	0.78-7.45	0.123	1.96	0.58-6.57	0.276
>10 & < = 15 cm	19.38	2.25-166.5	0.007	1.71	0.43-6.71	0.439
>15 cm	2.7	0.58-13.16	0.2	4.57	1.18-17.68	0.028
						
**Tumor Location**						
Upper limb	1			1		
Lower limb	4	1.05-15.2	0.042	9	1.05-77.03	0.045
Thorax	1.6	0.37-6.8	0.525	3.4	0.34-34.99	0.299
Head & neck	1.6	0.08-31.7	0.758			
Retroperitoneum	9.6	1.48-62.15	0.018	16	1.6-159.3	0.018
						
**Plane of Tumor**						
Subcutis	1			1		
Muscular plane	4.14	1.3-13.2	0.016	4.01	1.31-12.32	0.015
Body cavity	8.05	1.62-39.8	0.011	5.6	1.6-19.6	0.007
						
**Circumscription**						
No	1			1		
Yes	0.2	0.07-0.55	0.002	1.005	0.40-2.5	0.991
						
**Necrosis**						
No	1			1		
Yes	18.13	2.28-143.6	0.006	4.98	1.7-14.3	< 0.001

**Table 4:** Clinicopathologic characteristics and expression of STAT3 in malignant soft tissue tumors.

Clinicopathological Characteristics	STAT3		
	**Negative(%)**	**Positive(%)**	**P-value**
	
**Number of patients**	2 (4.17)	46 (95.83)	
			
**Tumour Size**			
< = 5 cm	0(0.00)	13(100.00)	0.537
>5 & < = 10 cm	1(8.33)	11(91.67)	
>10 & < = 15 cm	0(0.00)	11(100.00)	
>15 & < = 20 cm	1(14.29)	6(85.71)	
>20 cm	0(0.00)	5(100.00)	
			
**Tumor Location**			
Upper limb	0(0.00)	5(100.00)	1
Lower limb	1(4.55)	21(95.45)	
Thorax	0(0.00)	7(100.00)	
Head & neck	0(0.00)	1(100.00)	
Retroperitoneum	1(7.69)	12(92.31)	
			
**Plane of Tumor**			
Subcutis	1(6.25)	15(93.75)	0.533
Muscular plane	0(0.00)	17(100.00)	
Body cavity	1(6.67)	14(93.33)	
			
**Circumscription**			
No	1(3.13)	31(96.88)	1
Yes	1(6.25)	15(93.75)	
			
**Capsulation**			
No	2(4.55)	42(95.45)	1
Yes	0(0.00)	4(100.00)	
			
**Necrosis**			
No	1(3.45)	28(96.55)	1
Yes	1(5.26)	18(94.74)	

#### Clinicopathological significance of STAT3 expression in soft tissue tumors

In our study, the expression of STAT3 in soft tissue tumors showed significant association with tumor size (OR = 19.38, 95% CI: 2.25-166.5, P = 0.003), tumor location (OR = 9.6, 95% CI:1.48-62.15, P = 0.025), plane of the tumor (OR = 8.05, 95% CI:1.62-39.8, P = 0.011), tumor circumscription (P = 0.005) and tumor necrosis (OR = 18.13, 95% CI: 2.28-143.6, P = 0.001). However, no significant association was observed between STAT3 expression with age group (P = 0.34) and tumor capsulation (P = 0.21).

#### Clinicopathological significance of pSTAT3 expression in soft tissue tumors

Expression of pSTAT3 in soft tissue tumors also exhibited significant association with tumor location (OR = 16, 95% CI: 1.6-159.3, P = 0.027), plane of tumor (P = 0.006) and tumor necrosis (OR = 4.98, 95% CI: 1.7-14.3, P = 0.002). However, pSTAT3 expression showed no significant association with age of the patients (P = 0.321), tumor size (P = 0.141), tumor circumscription (P = 0.991), and capsulation (P = 0.957).

## Discussion

STAT3 is a major mediator of tumorigenesis, and has been shown to be vital for tumor cell growth, proliferation, and apoptosis [10-12]. Constitutive activation of STAT3 has been documented in ovarian, breast, colon, prostate, and several other types of cancer [5,13-16]. Although the contribution of STAT3 to epithelial cancers and hematologic malignancies has been described in detail, little is known on the role of STAT3 dysregulation in sarcomas. The purpose of this study was to investigate the expression levels of STAT3 and pSTAT3 in various soft tissue tumors and to associate it with its clinicopathological characteristics. Our data suggests that STAT3 may be a key regulatory molecule in the malignant potential of soft tissue tumors and can be piloted as diagnostic marker in soft tissue tumors.

In the current study we observed a distinct pattern of STAT3 and pSTAT3 expression in soft tissue tumors, which differed significantly between benign, intermediate and malignant tumors and showed significant association with various histopathological parameters. Age group is not associated with STAT3 (P = 0.58) and pSTAT3 (P = 0.321) expressions. However, STAT3 and pSTAT3 expressions were significantly associated with grade of the tumor (P < 0.001). 46 out of the 48 malignant tumors (95.8%) and 6 out of the 9 intermediate tumors (66.7%) were STAT3 positive. Malignant tumors were 107.3 times more likely to express STAT3, when benign or intermediate tumor is the reference (OR = 107.3, 95% CI: 20.24-569). 24 out of the 48 malignant tumors (50%) and 4 out of the 9 intermediate tumors (44.4%) were pSTAT3 positive. Malignant tumors were 7.5 times more likely to express pSTAT3, when benign or intermediate tumor is the reference (OR = 7.5, 95% CI: 2.28-24.5). This is in agreement with the study by Chun *et al *[17], were it was observed that STAT3 signaling pathway is constitutively activated in rhabdomyosarcoma and osteosarcoma cells. It has been previously reported that STAT3 is overexpressed in cutaneous angiosarcoma, pyogenic granuloma, Ewing's sarcoma, Kaposi's sarcoma and in primary effusion lymphomas [18-20].

The other histopathological factors associated with STAT3 and pSTAT3 expressions were tumor location (P = 0.025, P = 0.027), plane of the tumor (P = 0.011, P = 0.006) and tumor necrosis (P = 0.001, P = 0.002). Out of 35 tumors in the lower extremities, 27(74.1%) were STAT3 positive and 15(42.9%) were pSTAT3 positive. 12 out of the 14 tumors in the retroperitoneum (85.7%) were STAT3 positive while pSTAT3 positives were 8(57.1%). Tumors in the retroperitoneum were more expressive of STAT3 (OR = 9.6, 95% CI: 1.48-62.15) and pSTAT3 (OR = 16, 95% CI: 1.6-159.3) when upper extremity is the reference. Tumor plane exhibited a positive trend with expression of STAT3 and pSTAT3, which were expressed in 51.16% and 18.6% of subcuitis, followed by the muscular plane (78.3% and 47.8%)) and body cavity (87.5% and 56.3%). Odds ratio for the muscular plane is 4.14 (95% CI 1.3-13.2) and body cavity is 8.05(1.62-39.8) for STAT3 expression. Odds ratio for muscular plane is 4.01(1.31-12.32) and body cavity is 5.6(1.6-19.6) for pSTAT3 when subcuitis as the reference. Out of the 21 tumors, which showed necrosis, 20 were found to be STAT3 positive (95.24%) and 13 were found to be pSTAT3 positive (61.9%). Tumors with necrosis were 18.13 times more likely to express STAT3 (OR = 18.13, 95% CI: 2.28-143.6) and 4.98 times more likely to express pSTAT3 (OR = 4.98, 95% CI: 1.7-14.3), when non-necrotic tumors are the reference.

In addition, tumor size also exhibited significant association with STAT3 expression (P = 0.003). Tumors greater than 10 cm and less than or equal to 15 cm in size were 19.38 times more likely to express STAT3 when tumors less than 5 cm is the reference (OR = 19.38, 95% CI: 2.25-166.5). We observed that tumors greater than 15 cm in size were 4.57 times more likely to express pSTAT3 when tumors less than 5 cm is the reference (OR = 4.57, 95% CI: 1.18-17.68). Significant association was observed between STAT3 expression and tumor circumscription (P = 0.001). Out of the 44 poorly circumscribed tumors 35 were STAT3 positive (79.55%). But pSTAT3 expression is not associated with tumor circumscription (P = 0.991). STAT3 and pSTAT3 expressions were not determined to associate with tumor capsulation (P = 0.21). However, whether STAT3 and pSTAT3 expression correlate with metastasis and recurrence needs to be evaluated.

The present study thus suggests that overexpression of STAT3 at the protein and gene level may be considered as a hallmark of sarcomas. Our data also indicates that increased activation of STAT3 could be associated with more aggressive biological behavior of soft tissue tumors. Although constitutive activation of STAT proteins is not the only contributing factor to transformation and cancer progression, its crucial role is still under investigation in soft tissue tumors. The mechanisms responsible for aberrant STAT activation in sarcomas remain uncertain and need further exploration. Moreover, knowledge of the cross-interaction of STAT molecules with other critical cellular proteins involved in growth regulation and survival may better serve to explain carcinogenesis in sarcomas.

## Conclusions

The overexpression of STAT3 and pSTAT3 (Tyr705) has been observed in human soft tissue tumor samples and the expression level increases with tumor grade progression. Our data showed that constitutive activation of STAT3 in human soft tissue tumors is significantly associated with its clinicopathological parameters such as tumor grade, plane of the tumor, tumor size and tumor necrosis, which may possibly have potential diagnostic and prognostic implications.

## Competing interests

The authors declare that they have no competing interests.

## Authors' contributions

AS and DD designed this study and carried out immnunohistochemistry staining, western blotting and RT-PCR and drafted the manuscript. LM, and KB, provided the clinical samples and collected clinical information and MR participated in the coordination of the study and helped to draft the manuscript. JV performed the statistical analysis. All authors read and approved the final manuscript.

## Supplementary Material

Additional file 1 **Table S1**. Clinicopathologic characteristics and expression of STAT3 and pSTAT3 in soft tissue tumors. Click here for file
