# Supplementary material for: Prognostic significance of STAT3 and phosphorylated STAT3 in human soft tissue tumors - a clinicopathological analysis
Source: J Exp Clin Cancer Res. 2011 May 16;30(1):56. doi: 10.1186/1756-9966-30-56 (PMC3105950; doi:10.1186/1756-9966-30-56)
Supplement: Additional file 1 — Table S1. Clinicopathologic characteristics and expression of STAT3 and pSTAT3 in soft tissue tumors. [file 1756-9966-30-56-S1.DOC]

**Table S1. Clinicopathologic characteristics and expression of STAT3 and pSTAT3 in soft tissue tumors**

**Clinicopathological**

**characteristics STAT3 pSTAT3**

Negative(%) Positive(%) Total P-value Negative(%) Positive(%) Total P-value

**Number of patients** 28(34.1) 54(65.8) 82(100) 54(65.8) 28(34.1) 82(100)

**Age**

<20 5(38.5) 8(61.5) 13(100) 0.58 9(69.2) 4(30.8) 13(100) 0.321

20-39 9(42.9) 12(57.1) 21(100) 17(81) 4(19.0) 21(100)

40-59 10(33.3) 20(66.7) 30(100) 17(56.7) 13(43.3) 30(100)

>=60 4(22.2) 14(77.8) 18(100) 11(61.1) 7(38.9) 18(100)

**Grade of tumor**

Benign 23(92) 2(8) 25(100) <0.001 25(100) 0(0) 25(100) <0.001

Intermediate 3(33.3) 6(66.7) 9(100) 5(55.6) 4(44.4) 9(100)

Malignant 2(4.2) 46(95.8) 48(100) 24(50) 24(50) 48(100)

**Tumor Size**

<=5 cm 18(58.1) 13(41.9) 31(100) 0.003 24(77.4) 7(22.6) 31(100) 0.141

>5 & <=10 cm 6(27.3) 16(72.7) 22(100) 14(63.6) 8(36.4) 22(100)

>10 & <=15 cm 1(6.7) 14(93.3) 15(100) 10(66.7) 5(33.3) 15(100)

>15 & <=20 cm 3(33.3) 6(66.7) 9(100) 5(55.6) 4(44.4) 9(100)

>20 cm 0(0) 5(100) 5(100) 1(20) 4(80) 5(100)

|  |
| --- |

**Tumor Location**

Upper limb 8(61.5) 5(38.5) 13(100) 0.0251 2(92.3) 1(7.7) 13(100) 0.027

Lower limb 8(22.9) 27(77.1) 35(100) 20(57.1) 15(42.9) 35(100)

Thorax 9(50) 9(50) 18(100) 14(77.8) 4(22.2) 18(100)

Head & neck 1(50) 1(50) 2(100) 2(100) 0(0) 2(100)

Retroperitoneum 2(14.3) 12(85.7) 14(100) 6(42.9) 8(57.1) 14(100)

**Plane of Tumor**

Subcutis 21(48.8) 22(51.2) 43(100) 0.0113 5(81.4) 8(18.6) 43(100) 0.006

Muscular plane 5(21.7) 18(78.3) 23(100) 12(52.2) 11(47.8) 23(100)

Body cavity 2(12.5) 14(87.5) 16(100) 7(43.7) 9(56.2) 16(100)

**Circumscription**

No 9(20.4) 35(79.5) 44(100) 0.005 29(65.9) 15(34.1) 44(100) 0.991

Yes 19(50) 19(50) 38(100) 25(65.8) 13(34.2) 38(100)

**Capsulation**

No 23(31.5) 50(68.5) 73(100) 0.151 48(65.7) 25(34.2) 73(100) 0.957

Yes 5(55.6) 4(44.4) 9(100) 6(66.7) 3(3.3) 9(100)

**Necrosis**

No 27(44.3) 34(55.7) 61(100) 0.001 46(75.4) 15(24.6) 61(100) 0.002

Yes 1(4.8) 20(95.2) 21(100) 8(38.1) 13(61.9) 21(100)
